# Enhancing 3D Printing Copper-PLA Composite Fabrication via Fused Deposition Modeling through Statistical Process Parameter Study

**DOI:** 10.3390/mi15091082

**Published:** 2024-08-27

**Authors:** Mahmoud Moradi, Omid Mehrabi, Fakhir A. Rasoul, Anas Abid Mattie, Friedemann Schaber, Rasoul Khandan

**Affiliations:** 1 Faculty of Arts, Science and Technology, University of Northampton, Northampton NN1 5PH, UK; friedemann.schaber@northampton.ac.uk; 2 Department of Mechanical Engineering, Esfarayen University of Technology, Esfarayen 96619-98195, Iran; omidmehrabi70@gmail.com; 3 Department of Air-Conditioning and Refrigeration Technical Engineering, College of Technical Engineering, Al-Kitab University, Kirkuk 36003, Iraq; 4 Manufacturing Technology Department, Duhok Technical Institute, Duhok Polytechnic University, Duhok 42001, Iraq; anas.mattie@dpu.edu.krd; 5 Faculty of Engineering and Science, University of Greenwich, Chatham ME4 4TB, UK; r.khandan@greenwich.ac.uk

**Keywords:** fused deposition modeling, Cu-PLA composite, mechanical properties, response surface method, analysis of variance

## Abstract

The rapid advancement of additive manufacturing (AM) technologies has provided new avenues for creating three-dimensional (3D) parts with intricate geometries. Fused Deposition Modeling (FDM) is a prominent technology in this domain, involving the layer-by-layer fabrication of objects by extruding a filament comprising a blend of polymer and metal powder. This study focuses on the FDM process using a filament of Copper–Polylactic Acid (Cu-PLA) composite, which capitalizes on the advantageous properties of copper (high electrical and thermal conductivity, corrosion resistance) combined with the easily processable thermoplastic PLA material. The research delves into the impact of FDM process parameters, specifically, infill percentage (IP), infill pattern (P), and layer thickness (LT) on the maximum failure load (N), percentage of elongation at break, and weight of Cu-PLA composite filament-based parts. The study employs the response surface method (RSM) with Design-Expert V11 software. The selected parameters include infill percentage at five levels (10, 20, 30, 40, and 50%), fill patterns at five levels (Grid, Triangle, Tri-Hexagonal, Cubic-Subdivision, and Lines), and layer thickness at five levels (0.1, 0.2, 0.3, 0.4, and 0.5 mm). Also, the optimal factor values were obtained. The findings highlight that layer thickness and infill percentage significantly influence the weight of the samples, with an observed increase as these parameters are raised. Additionally, an increase in layer thickness and infill percentage corresponds to a higher maximum failure load in the specimens. The peak maximum failure load (230 N) is achieved at a 0.5 mm layer thickness and Tri-Hexagonal pattern. As the infill percentage changes from 10% to 50%, the percentage of elongation at break decreases. The maximum percentage of elongation at break is attained with a 20% infill percentage, 0.2 mm layer thickness, and 0.5 Cubic-Subdivision pattern. Using a multi-objective response optimization, the layer thickness of 0.152 mm, an infill percentage of 32.909%, and a Grid infill pattern was found to be the best configuration.

## 1. Introduction 

The additive manufacturing process allows producing objects of intricate and customized shapes made up of internal structures that cannot be created through traditional manufacturing methods [1,2,3,4]. The Fused Deposition Modeling (FDM) method is a variant of additive manufacturing technology. The FDM technique uses a heated thermoplastic filament passing through a heated nozzle whose movements are guided by computer-generated G-codes to follow a designed path [5,6]. The molten filament is deposited in successive layers onto a build platform to construct three-dimensional (3D) objects. Many factors, including nozzle and bed temperature, printing speed, layer height (LH), raster angle (RA), infill pattern (P), infill percentages (IP), and width layer (WL), have a significant impact on the mechanical properties of 3D printed materials by FDM during the printing process [7,8,9,10].

While FDM has proven to be efficient and cost-effective for producing objects with thermoplastic materials (PLA, acrylonitrile butadiene styrene, ABS), the desire to augment the mechanical [11,12,13], electrical [14,15,16] and thermal attributes of FDM printed parts have foreseen the development of other composite filaments made out of a polymer matrix infused with additives such as metals, carbon fibers, or ceramics, imparting unique characteristics to the printed parts [17,18,19,20,21]. Luo et al. [22] produced multi-walled carbon nanotubes (MWCNT) and PLA Composite with the FDM method. They investigated the effect of carbon nanotube weight on the mechanical properties of the MWCNTs/PLA composite. In an investigation led by Bortoli et al. [23], carbon nanotubes (CNT) were used to improve the mechanical properties of PLA. The results show that adding CNT to PLA significantly increases the mechanical characterization of samples. Jain et al. [24] compared the elongation at break, modulus, and tensile strength of carbon fiber-poly lactic acid (CF-PLA), graphene-PLA (Gr-PLA), and carbon nano tubes-PLA (MWCNTs-PLA) composites fabricated by fused filament fabrication (FFF). 

Integrating copper particles into the PLA matrix produces a composite filament that exhibits improved mechanical strength, electrical conductivity, and thermal performance compared to pure PLA [25]. Incorporating copper particles into the PLA matrix results in a commendable performance, rendering Cu-PLA appealing for applications that demand improved properties. This includes use in functional prototypes, electrical components, and heat sinks [26]. The effect of different raster angles on the mechanical characterization of PLA and Cu-PLA composite produced by the FDM method has been studied [27]. The results indicate that the maximum tensile strength and dynamic mechanical properties of PLA and Cu-PLA composite were obtained at a raster angle of 0°. Kottasamy et al. [28] used response surface methodology (RSM) to evaluate the impact of infill patterns (i.e., rectilinear, grid, concentric, octagram-spiral, and honeycomb) on the mechanical properties of Cu-PLA composite. They demonstrated that the maximum values of the yield strength and ultimate tensile strength were 12.8 and 25.20 MPa, respectively, at the concentric infill pattern. Additionally, Kottasamy et al. [29] optimized the impact strength of Cu-PLA fabricated by FDM. Using the RSM method, they analyzed the influence of Cu wt.% and infill pattern on the impact strength. Balamurugan et al. [30] investigated the influence of bed and nozzle temperature on the compressive and flexural strength of PLA/Cu specimens. The Taguchi method was employed by researchers [31] to assess the impact of raster angle, nozzle diameter, infill percentages, extruding speed, and extruder temperature on the tensile strength (UTS) of PLA.

According to Pavan et al. [32], the rise in bed and nozzle temperatures imports the brittle feature of Cu-PLA composite fabricated by FDM. In addition, flexural strength is significantly impacted by layer height. Kesavarma et al. [33] produced Cu-PLA composite with 25 wt.% and 80 wt.% of Cu with various infill patterns. The sample with a concentric infill pattern and a 25-weight percent Cu composition attained a flexural strength of 25.98 MPa. Vu et al. [34] investigated the mechanical and thermal properties of PMMA20-Cu/PLA and Cu/PLA composites with different weight percentages of copper produced by the FDM method. It was found that the PLA composites that consisted of 20-weight percent PMMA and 40-weight percent Cu had a thermal conductivity of 0.49 W m^−1^ K^−1^, which was greater than pure PLA. Research conducted by Yang et al. [35] found that increasing the infill percentages significantly expands Cu-PLA composites’ ultimate strength and stiffness. The influence of build orientations, raster angles, and various PLA composites (i.e., wood, ceramics, copper, aluminum, and carbon fiber) on the mechanical properties (tensile and flexural characteristics) has been evaluated [36]. The results reveal that adding ceramic, copper, and aluminum to PLA will improve the UTS compared with pure PLA samples. The maximum UTS and modulus occur at a +45°/−45° raster angle [36]. Using the Taguchi technique, Palaniyappan et al. [37] evaluated how layer height, printing temperature, and infill density affected tensile strength/density. The results suggested that at a layer height of 0.1 mm, an extrusion temperature of 215 °C, and an infill density of roughly 80%, the tensile strength/density has been discovered to be higher.

The production of Cu-PLA composite using the FDM method is a novel topic that has garnered significant attention recently. While the potential benefits of employing Cu-PLA composite filaments in FDM are evident, optimizing critical 3D printing process parameters for this material poses a challenge. To facilitate the actual application of Cu-PLA composite in the industry, there is a need for a systematic comprehension of how these parameters impact the process and the ensuing properties of printed parts, aiming to achieve consistent and desirable outcomes. An extensive literature review revealed that while numerous studies have analyzed the mechanical properties of Cu-PLA composite materials fabricated by FDM machines, no prior research has focused on statistical modeling and optimizing the FDM process factors (i.e., layer thickness, infill percentage, and fill pattern) that affect the maximum failure load, percentage of elongation at break, and part weight of PLA/Cu composites fabricated using the FDM method. Therefore, this paper seeks to assess the impact of infill percentage, fill pattern, and layer thickness on the maximum failure load (N), percentage of elongation at break, and weight of printed Cu-PLA composites. The evaluation is conducted by applying response surface methodology (RSM) to understand the correlation between the parameters. Finally, the optimal printed conditions are proposed to obtain minimum part weight, maximum failure load, and percentage of elongation at break for Cu-PLA composite specimens.

## 2. Materials and Methods 

### 2.1. Design of Experiments

**Figure 1:**
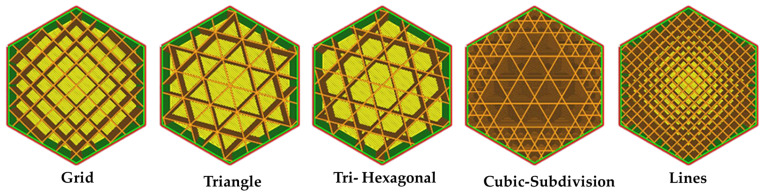
Infill patterns were used in the FDM process for this research.

Design of experiments is a systematic, efficient method to investigate the relationship between independent input variables and output responses [38,39,40,41]. This work was conducted by designing an experiment using a response surface method (RSM). The RSM method saves cost, time, and resources by reducing the number of experiments. Furthermore, RSM creates a predictive model of the system with a high level of accuracy. Additionally, the interaction effects of each input factor, the regression model, and the impact of each factor on responses can be easily extracted using RSM [42,43]. Design Expert v11 software was used for the RSM design experiment. Each parameter was investigated at five levels to evaluate the influence of infill percentage, fill pattern, and layer thickness. These levels were selected based on several pretests. The studied parameters and levels are shown in Table 1. The FDM process parameters and their levels were selected based on several pretest experiments and the literature review results. By utilizing the levels of take-into-consideration parameters (Table 1), the experimental design was created by CCD, which included 17 experiments. A central point (infill percentage: 30%, pattern: Tri-Hexagonal, and layer thickness: 0.3 mm) was replicated three times to evaluate the Lack of Fit and to estimate any experimental error. Figure 1 presents the infill patterns used in the FDM process for this work. In Table 2, the input variables and output variables were reported.

### 2.2. Experimental Work

**Figure 2:**
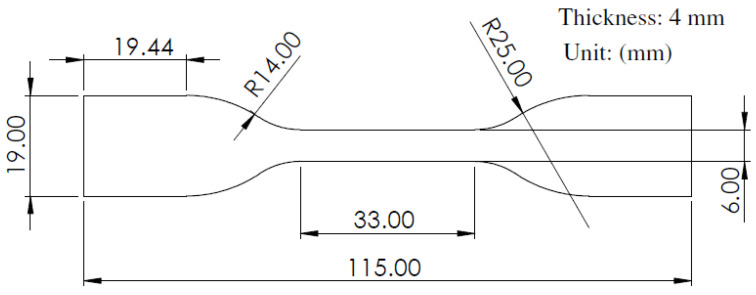
Geometry of tensile specimens according to ASTM D638 Type IV [44].

**Figure 3:**
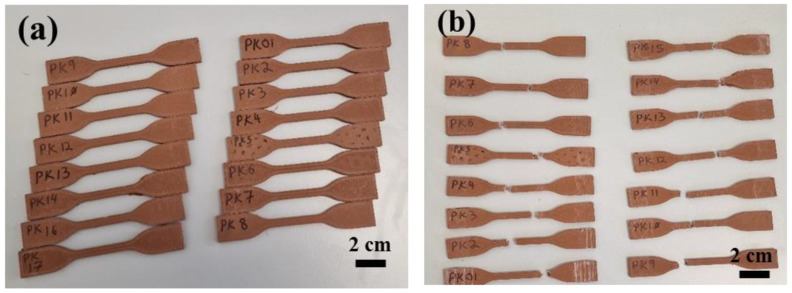
(**a**) 3D printed Cu-PLA composite specimen samples, (**b**) specimens after tensile test.

The Ultimaker Cura FDM 3D printer (Utrecht, The Netherlands), with the interface software for filament extrusion and printing, was used to print specimen samples. Firstly, a CAD model according to ASTM D638 Type IV standard [44] was created utilizing Solidworks v2022 software. Figure 2 presents the geometry of the tensile specimens according to ASTM D638 Type IV. This work is a composite filament made of copper and polylactic acid (Cu-PLA). The filament comprises biodegradable PLA, evenly dispersed spheroidized shape, a diameter ranging from 30 to 50 µm, and around 80% copper particles infused in a PLA matrix. Seventeen Cu-PLA composite specimens were fabricated utilizing the FDM technique suggested by the Design Expert v11 software, according to Table 2. The fixed parameters of the FDM process were determined based on a series of preliminary tests. For this purpose, the print temperature, bed temperature, and printing speed were set to 195 °C, 60 °C, and 40 mm/s, respectively. Figure 3a illustrates the 3D-printed Cu-PLA composite samples.

After printing the Cu-PLA composite specimen according to ASTM D638 Type IV, the mass of each printed component was determined by weighing the samples using a calibrated scale. Then, an Instron 5567 universal tensile testing machine equipped with a 5 kN load cell was used to conduct the tensile test. Under a controlled test environment (20–22 °C and indoor humidity between 30% and 60%), a constant load speed of 1 mm/min was applied (ASTM D638). Tension was constantly applied to each specimen sample until it fractured, as shown in Figure 3b.

## 3. Results and Discussion

The study’s output responses were considered the maximum failure load (N), percentage of elongation at break, and weight of the printed Cu-PLA composite. The influence of the input variables on the output responses was examined using the Analysis of Variance (ANOVA).

### 3.1. Maximum Failure Load

The ANOVA results for the maximum failure load (see Table 3) show that IP, LT, and IP² are the effective parameters influencing the maximum failure load. The *p*-value is the most significant parameter that is essential for interpretation. The matching parameter is statistically significant if the *p*-value is less than the significance level of 0.05. In addition, the interaction terms are not significant. The model for maximum failure load is significant (*p*-value = 0.0015) and Lack of Fit is insignificant (*p*-value = 0.1569). Equation (1) shows a regression equation for maximum failure load Based on coded values. The design expert software suggested that forecasting maximum failure load is quadratic. The R-squared (86.63%) and Adj R-squared (94.15%) illustrate the suitable regression model.

(1) $${\left(Maximum\;failure\;load\;\right)}^{2.34}=281,132+22,679.6\;A-28,340.7\;B+89,639.8\;C-43,126.2\;A\times B+\;14,289.5\;A\times C+55,963.8\;B\times C-83,659.9\;A^{2}-12,072.2\;B^{2}+-34,023.9\;C^{2}$$

**Figure 4:**
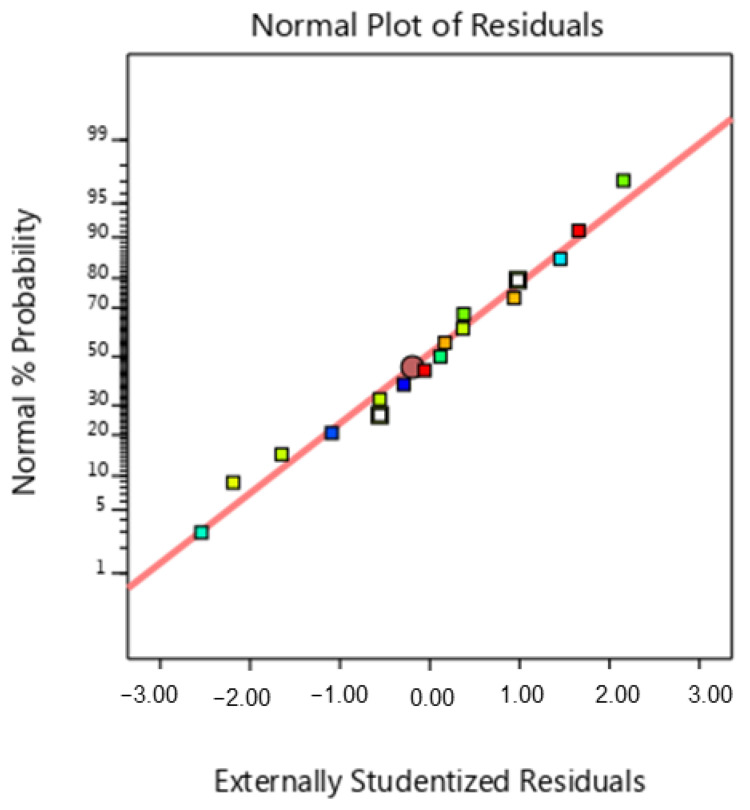
Normal probability diagram for maximum failure load. (Colored squares are distributions of the data from the trend line and the middle red circle represents the mean of normal data).

**Figure 5:**
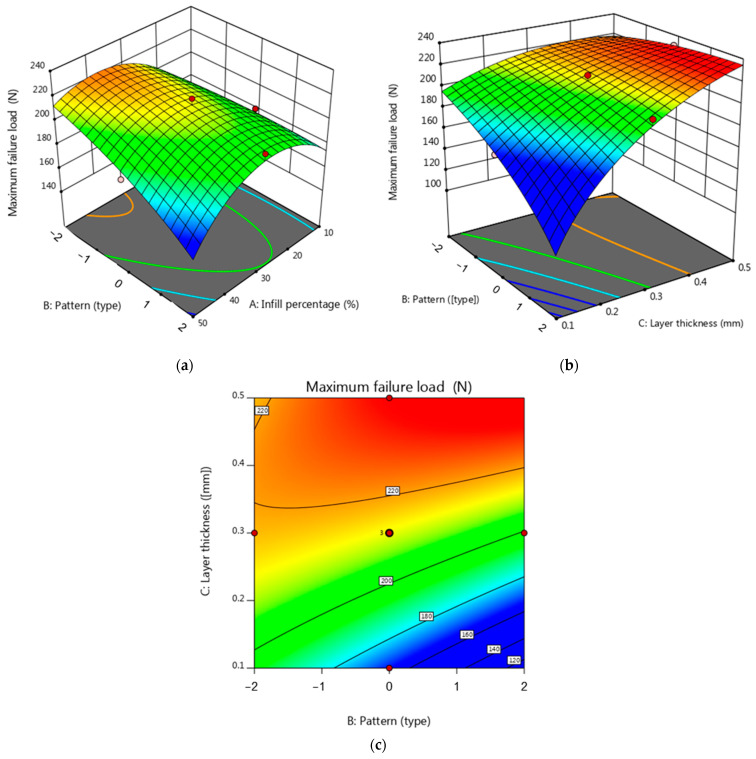
(**a**) 3D surface plot for maximum failure load (in terms of pattern and infill percentage), (**b**) 3D surface plot for maximum failure load (in terms of fill pattern and layer thickness), (**c**) contour plot for maximum failure load (in terms of pattern and layer thickness).

The Analysis of Variance results are statistically valid when the variances are equal and the data have a normal distribution. These assumptions were verified by examining a normal plot of residuals for maximum failure load (see Figure 4). Figure 4 shows that the residuals closely match the line. This indicates that the residuals have a normal distribution. Figure 5a demonstrates the interaction influence of the infill pattern and infill percentage on the maximum failure load. As seen in Figure 5a, it is clear that the maximum failure load increased by decreasing the infill percentage. However, it is clear from the slope of the curve that the influence of the infill percentage on maximum failure load is higher than the influence of the infill pattern. Infill density plays a crucial role in determining the strength of the printed specimens, regardless of the filling pattern or print orientation. The infill percentage affects the amount of material deposited within the structure of the samples. More material is injected into the object’s interior as the infill percentage increases, which increases structural density. This increased structural density raises the object’s maximum failure load by strengthening its load-bearing capability. This augmented density enhances the load-bearing capacity of the samples, resulting in a higher maximum failure load.

Furthermore, as shown in Figure 5b, the maximum failure load increases when the layer thickness increases. Based on both Figure 5b,c, the lowest maximum failure load occurs at the maximum level of the pattern (Lines pattern). In this study, the specimens printed with the cubic pattern exhibited higher strength values than the linear samples. This can be attributed to the more excellent adhesion and improved bonding between layers facilitated by the increased contact surface area. As a result, the cubic pattern demonstrated enhanced load distribution and improved overall performance.

**Figure 6:**
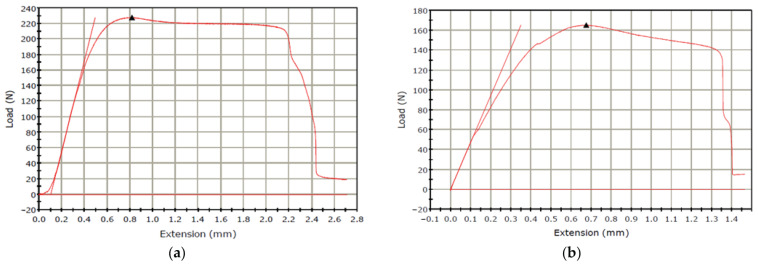
(**a**) Samples #6 with lowest maximum failure load (infill percentage: 30%, fill pattern: Tri-Hexagonal and layer thickness: 0.1 mm), (**b**) Samples #5 with highest maximum failure load (infill percentage: 30%, fill pattern: Tri-Hexagonal and layer thickness: 0.5 mm).

**Figure 7:**
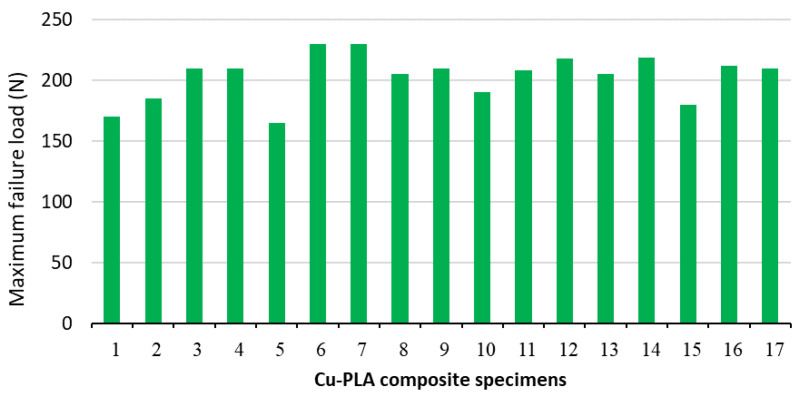
An overview of maximum failure load value for all printed Cu-PLA composite samples.

**Figure 8:**
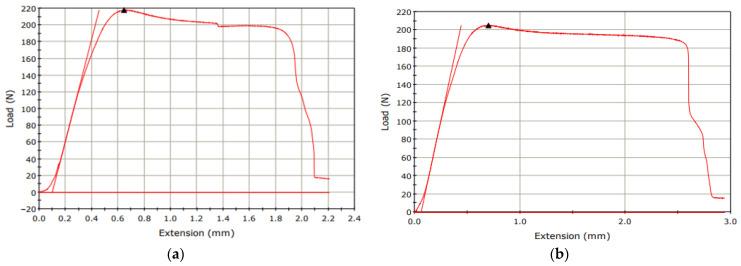
(**a**) Tensile test results of sample #12 (infill percentage: 30%, fill pattern: Tri-Hexagonal and layer thickness: 0.3 mm), (**b**) tensile tests of sample #13 (infill percentage: 30%, pattern: Lines and layer thickness: 0.3 mm).

Figure 6 presents the graph of load vs. extension in tensile tests of samples #6 and #5. As seen in Figure 6a and Figure 7, the highest maximum failure load (230 N) is obtained with a layer thickness of 0.5 mm, while the lowest maximum failure load (165 N) is obtained with a layer thickness of 0.1 mm (see Figure 6a and Figure 7). Also, the comparison of two tensile test results (load vs. extension) of two samples fabricated with a Lines pattern and a Tri-Hexagonal shows that the maximum failure load in the sample #12 fabricated with a Tri-Hexagonal pattern is 218 N (Figure 8a) and in the sample #13 manufactured with a Lines pattern is 205 N (Figure 8b).

### 3.2. Percentage of Elongation at Break

According to the results of an Analysis of Variance on the percentage of elongation at break (Table 4), layer thickness, the interaction IP × P, and the quadratic term of layer thickness have significance. The results also indicate that the interaction between IP × LT and P × LT and the quadratic terms of IP^2^ and P^2^ are insignificant. The regression model should be significant, and the Lack of Fit should be insignificant in the excellent analysis. The model for a percentage of elongation at break is significant (*p*-value = 0.0105) and Lack of Fit is insignificant (*p*-value = 0.5821). Based on coded values, Equation (2) shows the final regression model for % elongation at break. The design expert software suggested forecasting that the maximum failure load is a quadratic model. The R-squared and Adj R-squared for the regression model were 89.48% and 75.95%, respectively, illustrating that the regression model is suitable.

(2) $${\left(\%\;elongation\;at\;break\right)}^{0.45}=0.656796+-0.0313644A+0.00591589B-0.00726341C-\;0.135216\;A\times B+0.00397408\;A\times C+0.0222443\;B\times C-0.00436197\;A^{2}+0.0187089B^{2}+\;0.0745998\;C^{2}$$

**Figure 9:**
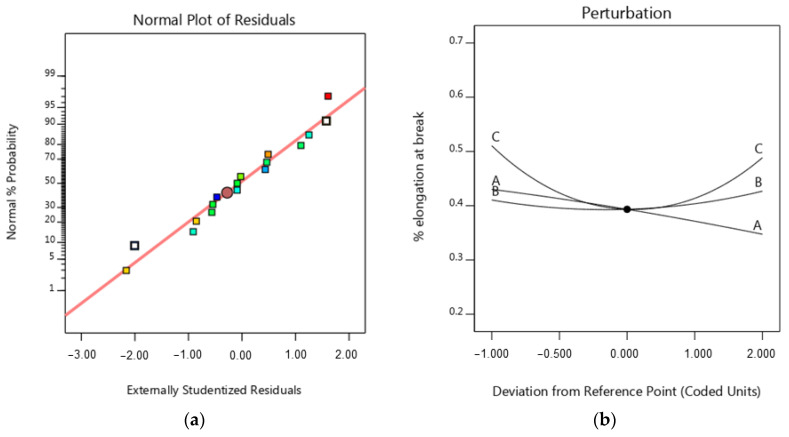
(**a**) Normal probability diagram for the percentage of elongation at break, (**b**) perturbation for the percentage of elongation at break (A = infill percentage, B = pattern, C = layer thickness).

**Figure 10:**
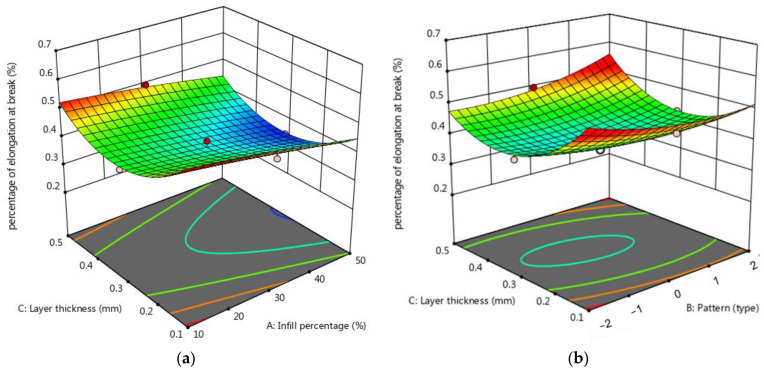
3D surface plot for the percentage of elongation at break (**a**) in terms of layer thickness and infill percentage, (**b**) in terms of layer thickness and pattern.

**Figure 11:**
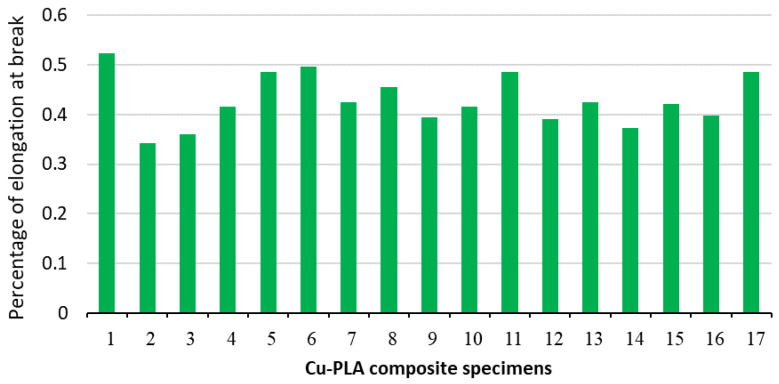
An overview of the percentage of elongation at break value for all printed Cu-PLA composite samples.

The normal diagram of residuals for a percentage of elongation at break, shown in Figure 9a, shows that the residuals dispersed along a straight line, and the errors exhibit a normal distribution on the normal probability diagram. Therefore, the regression model for the percentage of elongation at break is suitable. The perturbation plot of the IP, pattern and layer thickness on the percentage of elongation at break is shown in Figure 9b. The slope of the line in the perturbation plot shows that the effect of infill percentage on the percentage of elongation at break is linear. As the infill percentage increases, the percentage of elongation at break decreases due to the decrease in flexibility and the increase in resistance to deformation of the samples. On the other hand, the influence of pattern and layer thickness on the percentage of elongation at break is non-linear (see Figure 10b). Based on Figure 10a,b, increasing the layer thickness from 0.1 to 0.3 mm reduces the percentage of elongation at break. Then, by increasing the layer thickness from 0.3 to 0.5 mm, the percentage of elongation at break increases. The slope of the curve of Figure 10a,b shows that the influence of the infill percentage and layer thickness on the percentage of elongation at break is higher than the influence of the infill pattern. The minimum percentage of elongation at break (0.341) happens with a Tri-Hexagonal pattern, 50% infill percentage, and 0.5 mm layer thickness, and the maximum percentage of elongation at break (0.523) with 20% mm IP, 0.2 mm layer thickness, and 0.5 Cubic- Subdivision pattern. Figure 11 provides an overview of the percentage of elongation at break for all printed Cu-PLA composite samples. Figure 11 shows that the maximum percentage of elongation at break (0.5232) is obtained in specimen 1, while the minimum percentage of elongation at break (0.34146) is obtained in specimen 2.

### 3.3. Weight

Table 5 shows the analysis of variance (ANOVA) table for weight. According to Table 5, the infill percentage and layer thickness have a high significance level, while the pattern is insignificant. According to Table 5, the R-sq and Adj R-squared for the regression model were 96.97% and 95.04%, respectively, which illustrates that the model almost perfectly fits the data. It is clear that changes in the infill percentage and layer thickness have a more significant influence on weight than changes in pattern. This is also clear in relation to the coefficients found using Equation (1). The design expert software suggested forecasting sample weight is a linear model. The infill percentage and layer thickness coefficients in Equation (1) are 25.25 and 69.52, respectively, while the coefficient of the fill pattern is 2.93. This shows that the impact of infill percentage, layer thickness, and pattern on weight varied significantly.

(3) $${\left(Weight\;\right)}^{1.89}=165.402+25.2562\;A-2.93259\;B+69.5207C$$

**Figure 12:**
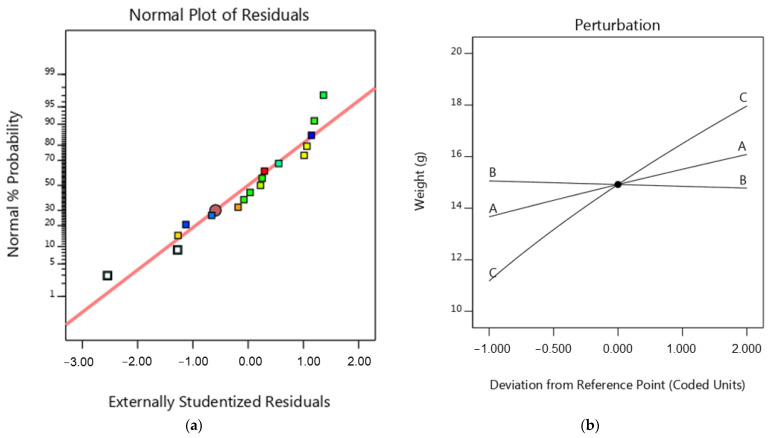
(**a**) Normal probability diagram for weight, (**b**) perturbation for the weight.

**Figure 13:**
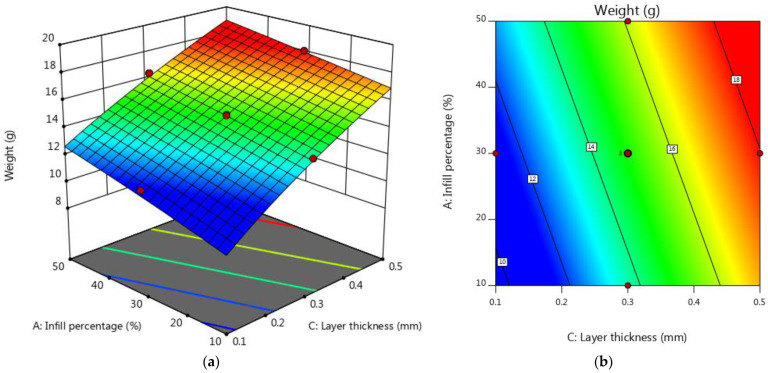
(**a**) 3D surface plot for the weight (in terms of IP and layer thickness) and (**b**) contour plot for the weight (in terms of IP and layer thickness).

The normal diagram of residuals for weight, shown in Figure 12a, indicates that the residuals closely match the graph. This suggests that the residuals have a normal distribution, supporting the regression model’s underlying assumptions. The perturbation plot of the FDM process parameters (i.e., IP, pattern, and LT) on the weight of the samples is shown in Figure 12b. As shown in Figure 12b, the weight is reduced by increasing the layer thickness. Increasing the thickness of the layers will make the layers denser during the FDM process. Thicker layers result in more material extruded per pass, and as a result, the weight of the samples increases. On the other hand, the thick layer increases the infill density and, as a result, increases the weight of the sample. In some cases, increasing LT in the FDM process can reduce porosity and empty space. This could lead to a denser part and, consequently, an increase in part weight. These results are consistent with those of previous studies [10,45]. The highest weight of the samples is achieved when the process is carried out at the highest layer thickness and infill percentage. Additively, the increase in infill percentage increases the weight of the sample; see Figure 12b and Figure 13a. Increasing the infill percentage of the material causes the samples to become more compact and increase the density, and as a result, this accumulates the weight of the samples. According to Figure 12a, the infill pattern has no considerable effect on the weight of the samples. As seen in Figure 13a,b, the minimum weight is obtained with an infill percentage of 10% and layer thickness of 0.1 mm.

**Figure 14:**
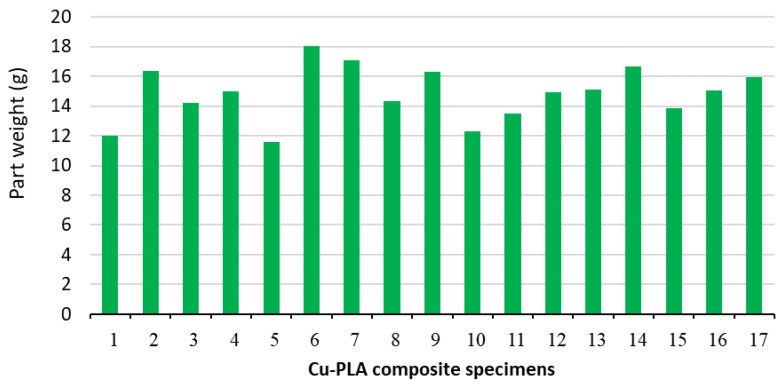
An overview of part weight value for all printed Cu-PLA composite specimens.

Figure 14 shows an overview of the part weight value for all printed Cu-PLA composite samples. It can be seen that the highest part weight of 18.0406 g is obtained in specimen 6 (IP = 30%, LT = 0.5 mm, and *p* = Tri- Hexagonal pattern), while the lowest part weight of 11.5934 g is obtained in specimen 6. (IP = 30%, LT = 0.1 mm, and *p* = Tri- Hexagonal pattern).

## 4. Optimization 

There are various methods available to optimize process parameters [46,47,48,49,50]. In this study, optimization of the FDM process parameters was performed using the RSM method with the assistance of Design Expert V11 software. According to Table 6, the criteria for optimization include minimum part weight, maximum failure load, and percentage of elongation at break for Cu-PLA composite specimens. A desirability value closer to 1 indicates a more ideal outcome. The highest desirability value was 0.810. From Table 7, the optimized parameter levels are a layer thickness of 0.152 mm, an infill percentage of 32.909%, and a Grid infill pattern, which results in a part weight of 12.617 g, a Maximum failure load of 205.999 N, and a percentage of elongation at break of 0.523. 

## 5. Conclusions

This paper explores the influence of three FDM process parameters—specifically, infill percentage, infill pattern, and layer thickness—on the maximum failure load (N), percentage of elongation at break, and weight of Cu-PLA composite specimens. The investigation has been conducted through both statistical analysis and experimental methods. The experimental design employed a response surface method. Subsequently, the key findings are presented:

The results show that the layer thickness and infill percentage are the most important parameters affecting the weight of samples. The weight of samples increases with the layer thickness and infill percentage increase.

The perturbation plot shows that specimens’ maximum failure load increases when layer thickness and infill percentage increase. On the other hand, the influence of pattern and layer thickness on maximum failure load is non-linear. The peak maximum failure load (230 N) occurs at 0.5 mm layer thickness, 30% infill percentage, and Tri-Hexagonal pattern, while the lowest maximum failure load (165 N) is obtained with 0.5 mm layer thickness.

As the infill percentage increases, the percentage of elongation at break decreases due to the decrease in flexibility and the increase in resistance to deformation of the samples. The effect of the infill percentage on the percentage of elongation at break is higher than the effect of the infill pattern and layer thickness.

The minimum percentage of elongation at break (0.341) occurs with a Tri-Hexagonal pattern, 50% infill percentage, and 0.5 mm layer thickness, and the maximum percentage of elongation at break (0.523) occurs with a 20% mm infill percentage, 0.2 mm layer thickness, and 0.5 Cubic-Subdivision pattern.

After response optimization, the minimum part weight, maximum failure load, and percentage of elongation at break were 12.617 g, 205.999 N, and 0.523, respectively. These values were obtained with settings of 32.909% IP, 0.152 mm LT, and Grid infill pattern.

## Figures and Tables

**Table 1:** Input variables for this study with their levels.

Variable	Notation	Unit	−2	−1	0	1	2
Infill percentage	IP	%	10	20	30	40	50
Fill Pattern	P	type	Grid	Triangle	Tri-Hexagonal	Cubic-Subdivision	Lines
Layer thickness	LT	mm	0.1	0.2	0.3	0.4	0.5

**Table 2:** Overview of the experimental results (weight, maximum failure load, percentage of elongation at break) for the input variables in Table 1.

NO	Input Variables						Output Variables		
	Coded Values			Actual Values			Weight (g)	Maximum Failure Load (N)	Percentage of Elongation at Break
	*IP* (%)	*P* (Type)	*LT* (mm)	*IP* (%)	*P* (Type)	*LT* (mm)			
#1	−1	1	−1	20	Cubic-Subdivision	0.2	12.004	170	0.5232
#2	2	0	0	50	Tri-Hexagonal	0.3	16.3556	185	0.34146
#3	0	0	0	30	Tri-Hexagonal	0.3	14.1908	210	0.35971
#4	0	0	0	30	Tri-Hexagonal	0.3	15.01	210	0.41526
#5	0	0	−2	30	Tri-Hexagonal	0.1	11.5934	165	0.4862
#6	0	0	2	30	Tri-Hexagonal	0.5	18.0406	230	0.49601
#7	1	−1	1	40	Triangle	0.4	17.0612	230	0.42429
#8	1	−1	−1	40	Triangle	0.2	14.3308	205	0.45558
#9	−1	−1	−1	20	Triangle	0.4	16.3048	210	0.39487
#10	−1	−1	−1	20	Triangle	0.2	12.3236	190	0.41526
#11	1	−1	−1	40	Cubic-Subdivision	0.2	13.4913	208	0.4864
#12	0	0	0	30	Tri-Hexagonal	0.3	14.9346	218	0.39094
#13	0	2	0	30	Lines	0.3	15.1088	205	0.42526
#14	1	1	1	40	Cubic-Subdivision	0.4	16.6574	219	0.37185
#15	−2	0	0	10	Tri-Hexagonal	0.3	13.845	180	0.42133
#16	0	−2	0	30	Grid	0.3	15.0386	212	0.39705
#17	−1	1	1	20	Cubic-Subdivision	0.4	15.9478	210	0.48588

**Table 3:** Analysis of Variance for input variables versus maximum failure load as the response.

Source	Sum of Squares	Degrees of Freedom	Mean Square	F-Value	*p*-Value	
Model	4.276 × 10^10^	9	4.751 × 10^9^	12.52	0.0015	significant
A-Infill percentage	1.825 × 10^9^	1	1.825 × 10^9^	4.81	0.0644	
B-Pattern	2.852 × 10^9^	1	2.852 × 10^9^	7.52	0.0288	
C-Layer thickness	2.851 × 10^10^	1	2.851 × 10^10^	75.14	<0.0001	
AB	7.423 × 10^8^	1	7.423 × 10^8^	1.96	0.2046	
AC	8.135 × 10^7^	1	8.135 × 10^7^	0.2144	0.6574	
BC	1.250 × 10^9^	1	1.250 × 10^9^	3.29	0.1124	
A²	8.463 × 10^9^	1	8.463 × 10^9^	22.31	0.0022	
B²	1.762 × 10^8^	1	1.762 × 10^8^	0.4645	0.5175	
C²	1.400 × 10^9^	1	1.400 × 10^9^	3.69	0.0962	
Residual	2.656 × 10^9^	7	3.794 × 10^9^			
Lack of Fit	2.205 × 10^9^	4	5.513 × 10^8^	3.67	0.1569	not significant
Pure Error	4.506 × 10^8^	3	1.502 × 10^8^			
Cor Total	4.542 × 10^10^	16				
R-sq = 94.15%			R-sq (Adjusted) = 86.63%			

**Table 4:** Analysis of Variance for input variables versus percentage of elongation at break as the response.

Source	Degrees of Freedom	F-Value	*p*-Value
Model	9	6.61	0.0105
A-Infill percentage	1	10.37	0.0147
B-Pattern	1	0.3692	0.5626
C-Layer thickness	1	0.5559	0.4802
AB	1	21.67	0.0023
AC	1	0.0187	0.8951
BC	1	0.5866	0.4688
A²	1	0.0683	0.8013
B²	1	1.26	0.2992
C²	1	19.99	0.0029
Residual	7		
Lack of Fit	4	0.8364	0.5821
Pure Error	3		
Cor Total	16		
R-sq = 89.48%		R-sq (Adjusted) = 75.95%	

**Table 5:** Analysis of Variance for input variables versus weight as response.

Source	Degrees of Freedom	F-Value	*p*-Value
Model	3	138.54	<0.0001
A-Infill percentage	1	47.89	<0.0001
B-Pattern	1	0.6261	0.4430
C-Layer thickness	1	362.84	<0.0001
Residual	13		
Lack of Fit	10	0.3611	0.9031
Pure Error	3		
Cor Total	16		
R- Squared = 96.97%		Adj R-Squared = 95.04%	

**Table 6:** Constraints and criteria for optimization.

Parameter/Response		Goal	Lower	Upper	Importance
Parameter	Layer thickness	In range	50	10	-
	Raster angle	In range	2	−2	-
	Infill percentage	In range	0.5	0.1	-
Response	Part weight	Minimize	18.0406	11.5934	3
	Maximum failure load	Maximize	230	165	3
	Elongation at break	Maximize	0.5232	0.34146	3

**Table 7:** Results of multi-objective response optimization.

Number	IP (%)	Pattern	LT (mm)	Weight	Maximum Failure Load	Percentage of Elongation at Break	Desirability	
1	32.909	−2.000	0.152	12.617	205.999	0.523	0.810	Selected
2	33.052	−2.000	0.153	12.642	206.182	0.524	0.810	
3	32.971	−2.000	0.150	12.588	205.773	0.526	0.810	
4	33.298	−2.000	0.146	12.527	205.292	0.533	0.809	
5	33.800	−2.000	0.143	12.506	205.061	0.540	0.809	

## Data Availability

The original contributions presented in the study are included in the article. Further inquiries can be directed to the corresponding author/s.
